# Supplementary figures and images for: Bradyrhizobium diazoefficiens USDA110 Nodulation of Aeschynomene afraspera Is Associated with Atypical Terminal Bacteroid Differentiation and Suboptimal Symbiotic Efficiency
Source: mSystems. 2021 May 11;6(3):e01237-20. doi: 10.1128/mSystems.01237-20 (PMC8125078; doi:10.1128/mSystems.01237-20)

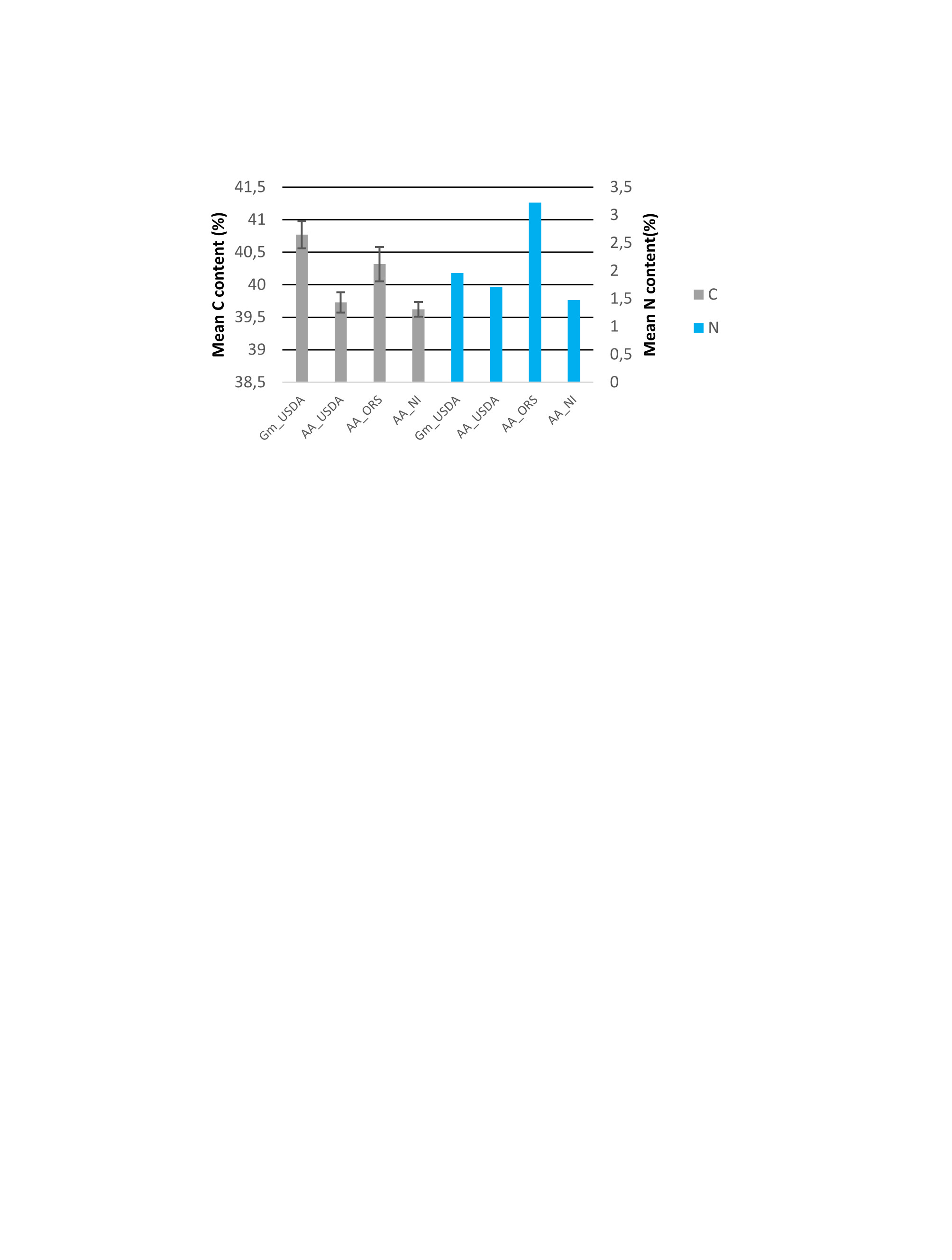

Supplement: FIG S1 [file mSystems.01237-20-sf001.tif]

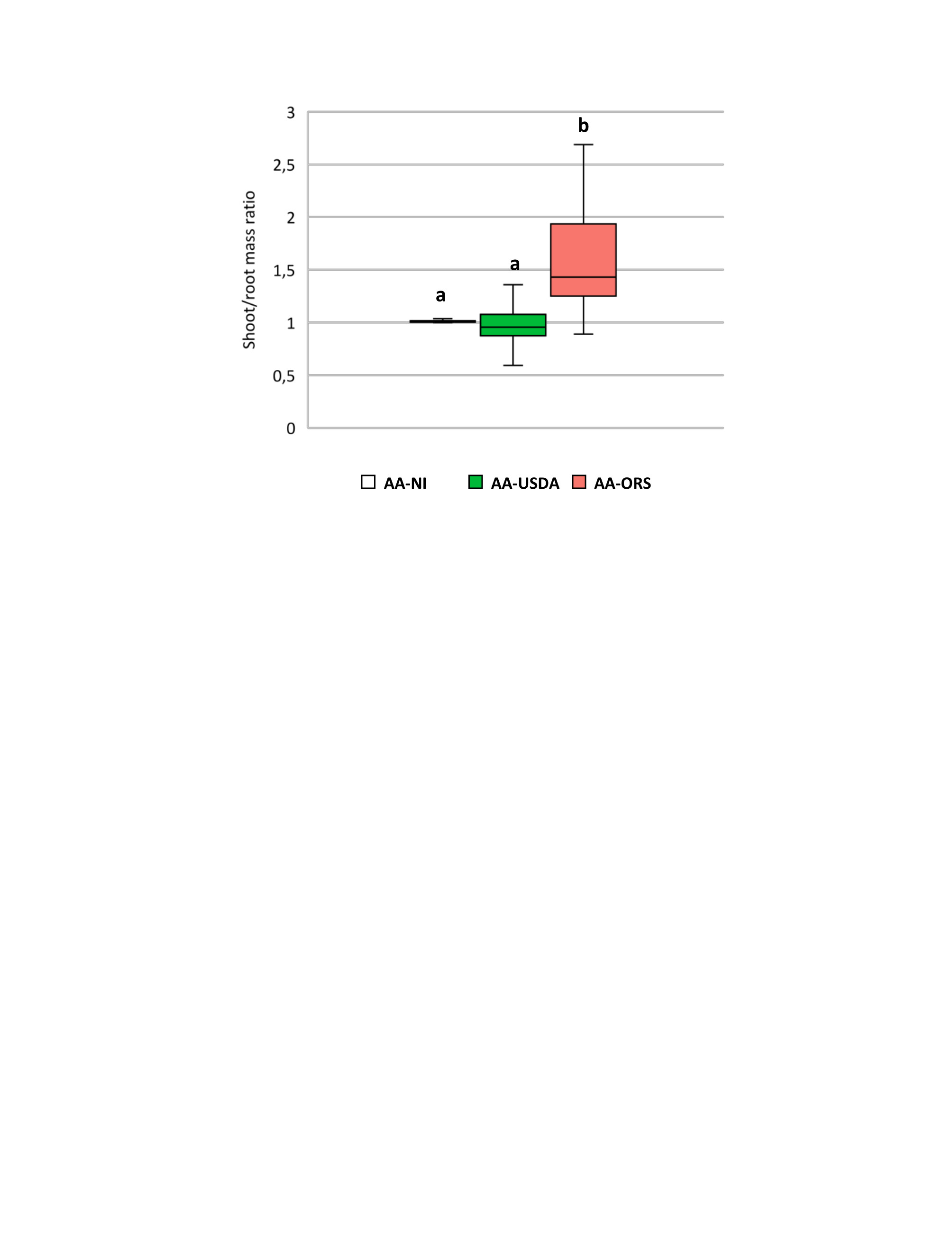

Supplement: FIG S2 [file mSystems.01237-20-sf002.tif]

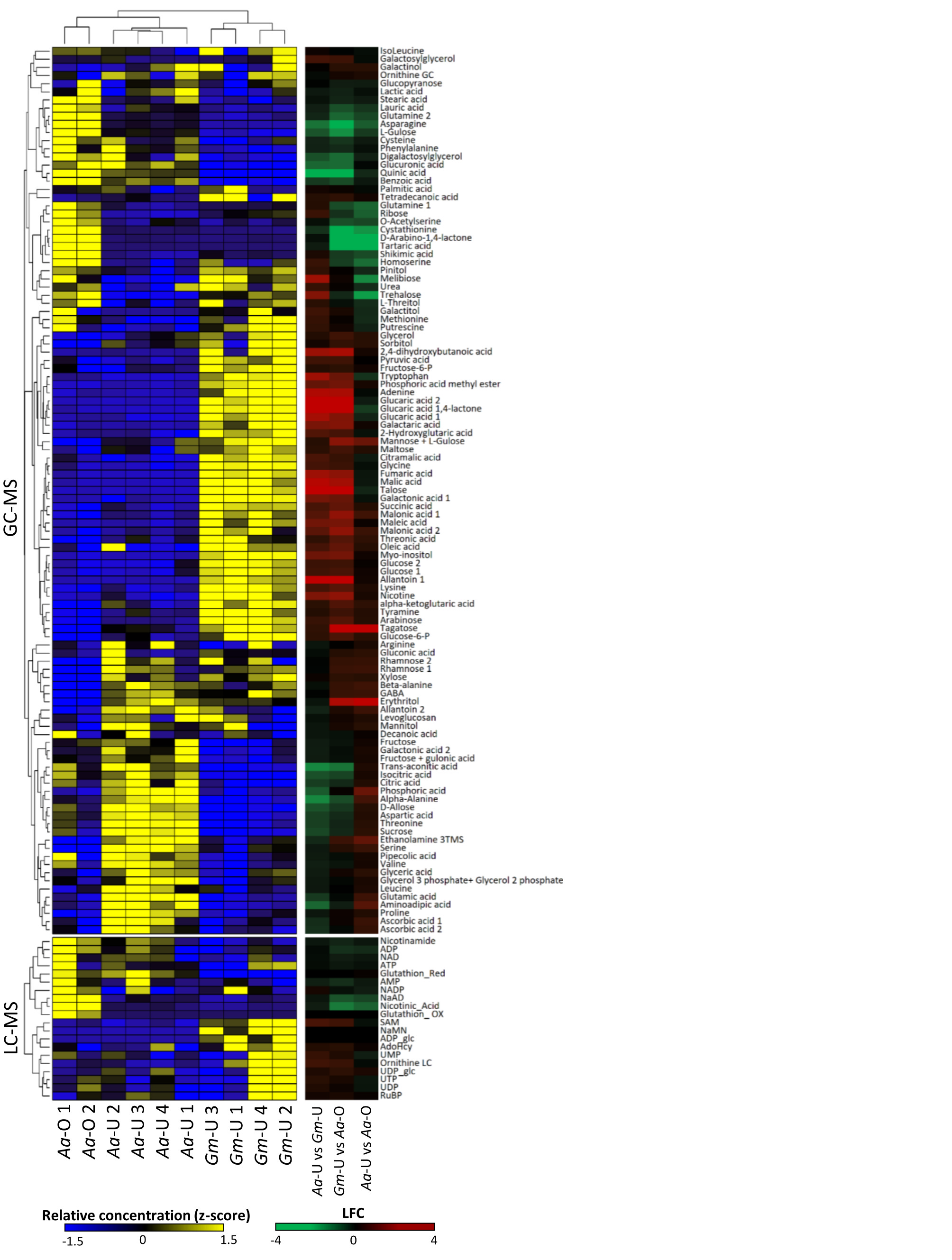

Supplement: FIG S3 [file mSystems.01237-20-sf003.tif]

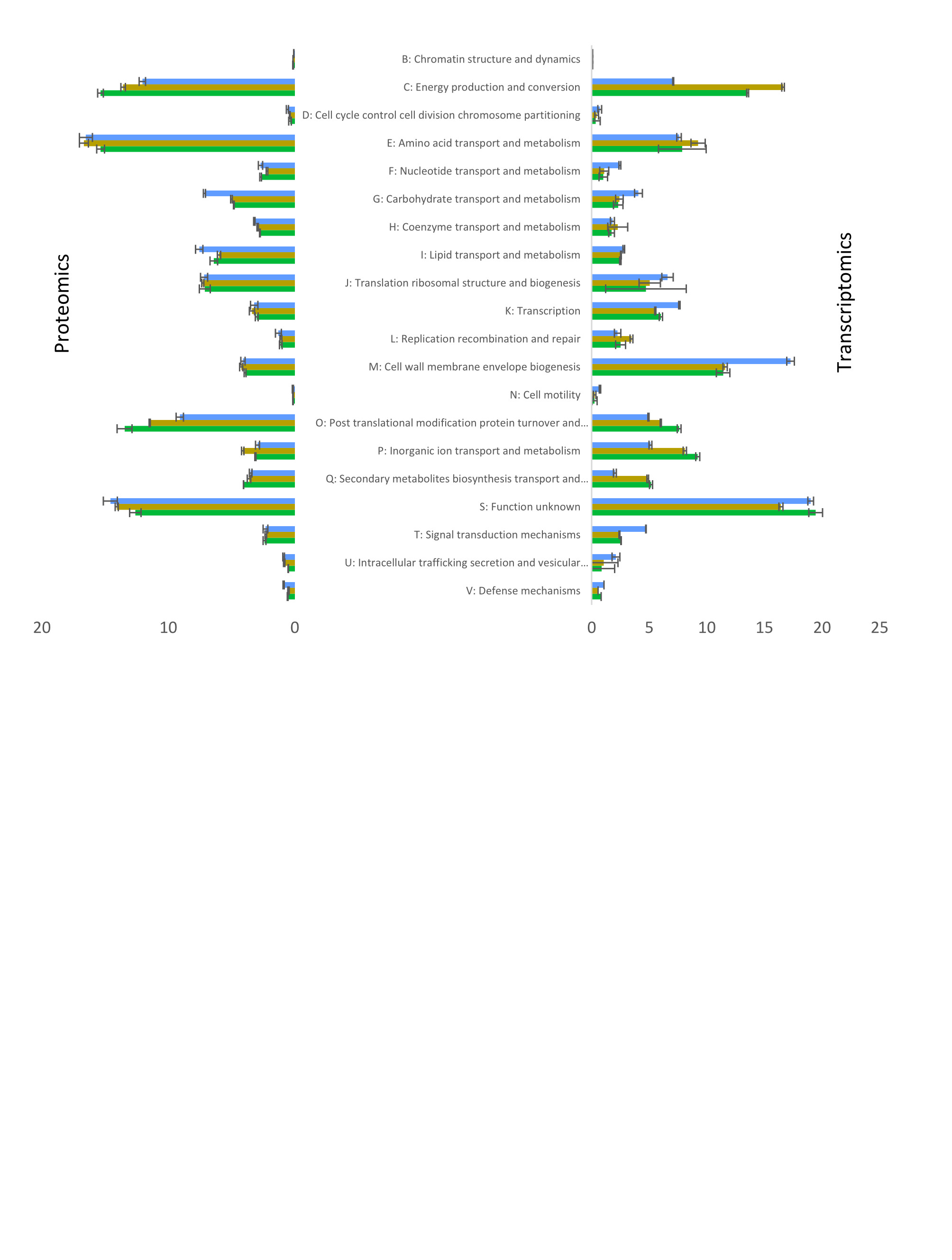

Supplement: FIG S4 [file mSystems.01237-20-sf004.tif]

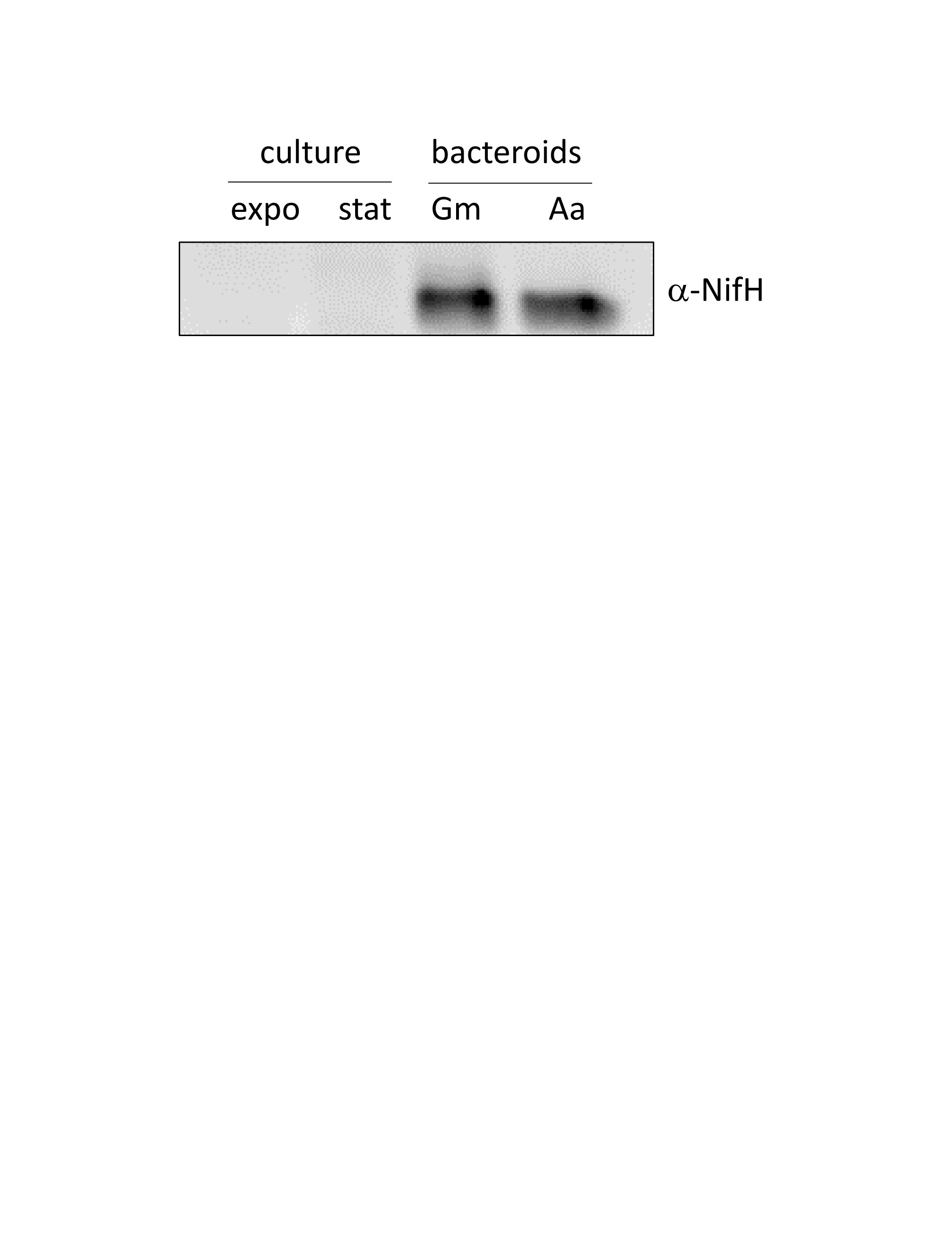

Supplement: FIG S5 [file mSystems.01237-20-sf005.tif]

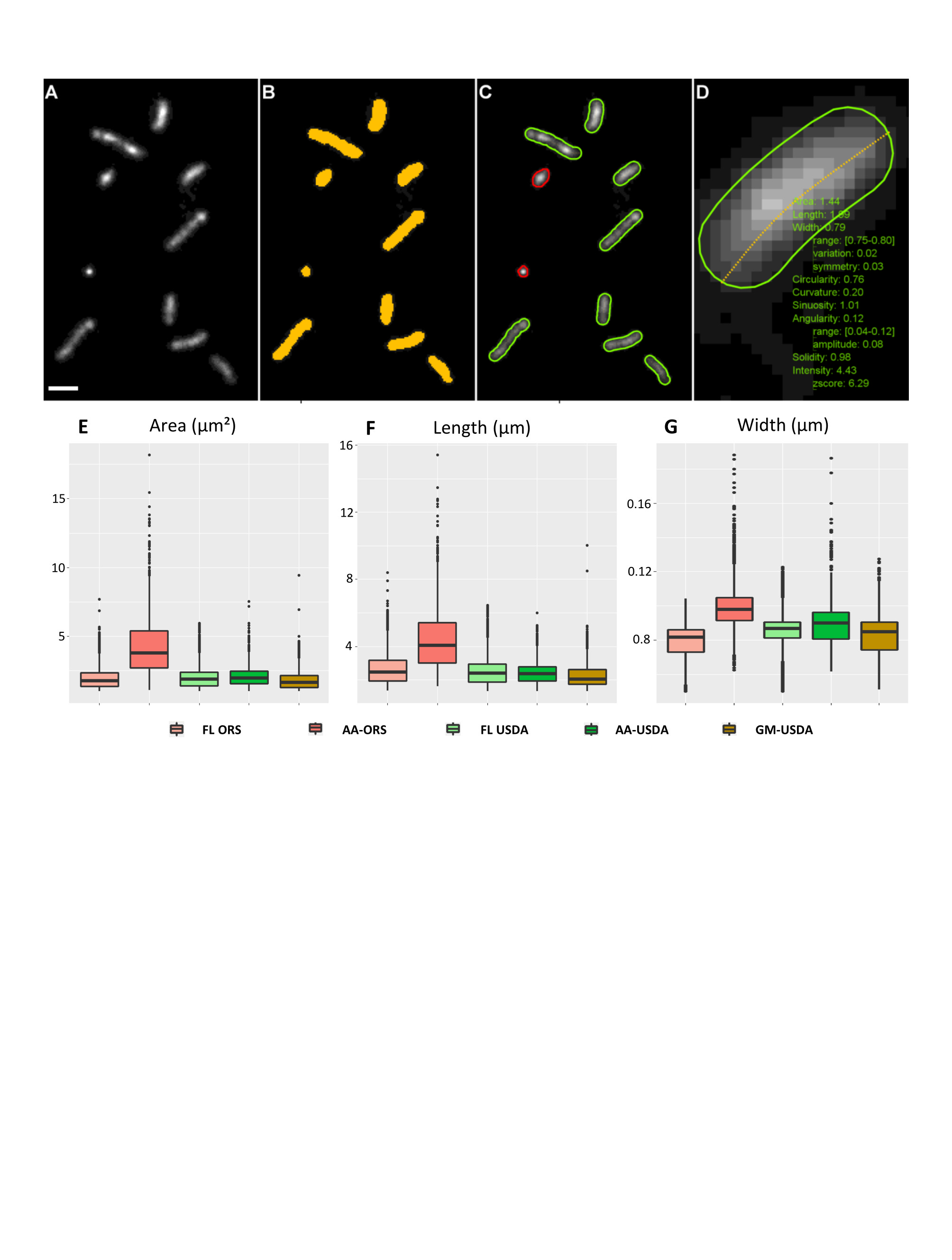

Supplement: FIG S6 [file mSystems.01237-20-sf006.tif]

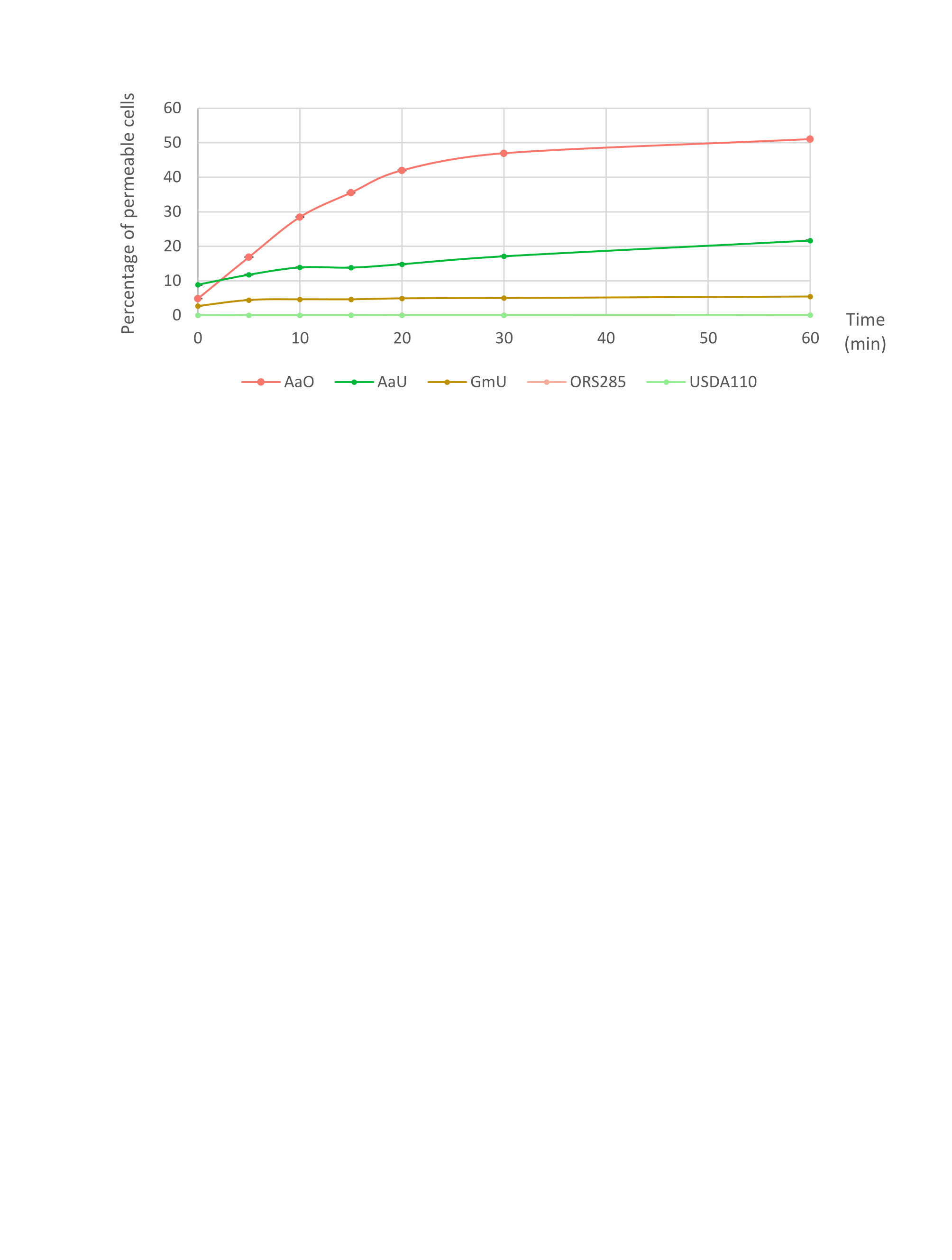

Supplement: FIG S7 [file mSystems.01237-20-sf007.tif]
